# Physician‐scientists in the United States at 2020: Trends and concerns

**DOI:** 10.1096/fj.202200327

**Published:** 2022-03-29

**Authors:** Howard H. Garrison, Timothy J. Ley

**Affiliations:** ^1^ Bethesda Maryland USA; ^2^ Section of Stem Cell Biology, Division of Oncology, Departments of Medicine and Genetics Washington University School of Medicine St. Louis Missouri USA

**Keywords:** physician‐scientist, research, training, workforce

## Abstract

Physician‐scientists comprise a unique and valuable part of the biomedical workforce, but for decades there has been concern about the number of physicians actively engaged in research. Reports have outlined the challenges facing physician‐scientists, and programs have been initiated to encourage and facilitate research careers for medically trained scientists. Many of these initiatives have demonstrated successful outcomes, but there has not been a recent summary of the impact of the past decade of effort. This report compiles available data from surveys of medical education and physician research participation to assess changes in the physician‐scientist workforce from 2011–2020. Several trends are positive: rising enrollments in MD‐PhD programs, greater levels of interest in research careers among matriculating medical students, more research experience during medical school and rising numbers of physicians in academic medicine, and an increase in first R01 grants to physician‐scientists. However, there are now decreased levels of interest in research careers among graduating medical students, a steady decline in MDs applying for NIH loan repayment program support, an increased age at first R01 grant success for physicians, and fewer physicians reporting research as their primary work activity: all of these indicators create concern for the stability of the career path. Despite a recommendation by the Physician‐Scientist Workforce in 2014 to create “real‐time” reporting on NIH grants and grantees to help the public assess trends, this initiative has not been completed. Better information is still needed to fully understand the status of the physician‐scientist workforce, and to assess efforts to stabilize this vulnerable career path.

**Abbreviations**
AAMC: American Association of Medical Colleges
AMA: American Medical Association
FASEB: Federation of American Societies for Experimental Biology
GME: Graduate Medical Education
LRP: Loan Repayment Program
NIH: National Institutes of Health
PSTP: Physician‐Scientist Training Program

## 1 INTRODUCTION

Physician‐scientists, by virtue of their understanding of both science and medicine, are able to ask clinically relevant questions in research settings, and incorporate scientific inquiry into the care of their patients. They bring a unique perspective to biomedical research.^1^ Dickler et al.^2^ found that 67% of the National Institutes of Health (NIH) grantees with an MD degree were pursuing clinical research (defined as research using humans or human tissue) compared to 43% of those with MD‐PhDs and 39% of those with PhDs. In a 2013 study, NIH grants to investigators with MD degrees were twice as likely to include human subjects as grants awarded to researchers with PhD degrees.^3^ Investigators with clinical training contribute essential knowledge and skills to research that leads to advances in medical practice. Physician‐scientists have received a large fraction of the major awards for biomedical research, including 37% of the Nobel Prizes in Physiology or Medicine, 41% of the Lasker Awards for basic science, and 61% of the Lasker Awards for clinical science.^4^ Goldstein and Brown^5^ described a “Golden Era of Nobel Laureates” from 1964–1972, when nine future Nobel Prize winners, all physician‐scientists, were trained at the NIH.

Maintaining the supply of these highly trained investigators, however, has been challenging. In 1979, Wyngaarden^6^ called physician‐investigators an endangered species, pointing to declining numbers of physicians applying for NIH training or research grants. Two decades later, Rosenberg^7^ documented a drop in number of first‐time MD applicants for NIH grants, which fell from 838 in 1994 to 575 in 1997 (but which later rebounded and stabilized). Zemlo et al.^8^ found that medical students' intentions to pursue research careers decreased substantially during the 1990s, while average debt levels of new medical school graduates steadily increased. In addition, the number of MDs supported on NIH training and fellowship grants declined, and, while the total number of applications for NIH research funding was growing, the number of first‐time grant applications submitted by MDs failed to increase. In 2004, after five years of major funding increases that doubled the NIH budget, NIH R01 applications from MDs rebounded from previous lows, but the growth rate was below that of PhD applicants. Moreover, MDs were significantly less likely than PhDs to submit applications for a second R01 grant, regardless of whether they were successful or not in their first attempt.^2^

In 2005, Ley and Rosenberg^9^ found some encouraging trends as the newly created NIH career development awards and loan repayment programs attracted a substantial number of early career MDs. They cautioned, however, that these optimistic outcomes needed to be closely monitored because their long‐term impact on the career pathway could not yet be assessed. Extending the analysis of physician‐scientists' careers to include data though 2011, Garrison and Deschamps^10^ found that the long‐term decline in the number of physicians entering research careers was temporarily halted during the 1998–2003 period of substantial NIH budget growth. But in subsequent years, when NIH budgets failed to keep pace with rising research costs, the research participation of physicians once again declined, relative to PhDs.

In its 2014 analysis, the NIH Physician‐Scientist Workforce Working Group found that the number of physician‐scientists had remained constant over the previous four decades, but their percentage in the research workforce was declining.^4^ The average age of physician‐scientists in the research workforce, moreover, was increasing and could lead to a marked decline of physician researchers when the current cohort retired. This report recommended more complete and transparent monitoring, and greater investment in the early career development of physician‐scientists.

Observers have also called attention to shortfalls of physician‐scientists in other countries. Traill et al.^11^ found a steep decline in the number of physician‐led projects funded by the Australian National Health and Medical Research Council, concluding that the Australian physician‐scientist is at risk. In response to diminished support for physician‐scientists, a consensus conference was convened in Canada to develop recommendations for more training and early career support.^12^

Wyngaarden^6^ attributed the shortfall in physician‐scientists to greater societal emphasis on medical care for underserved populations, instability of federal research funding, medical school curriculum revisions, changes in requirements for board certification, and the payback provisions of the National Research Service Award. Gill^13^ ascribed the decline in physician‐scientists' participation in biomedical research to economic and intellectual changes that made research careers less attractive for young physicians, while Moody^14^ pointed to cost‐containment policies limiting opportunities for research in clinical settings. The growing debt burden of medical school graduates, increased length of postdoctoral training required for a successful research career, instability inherent in an NIH‐funded research career, and the explosive growth of managed care were identified by Rosenberg as potential reasons for a decline in physician‐scientists.^7^

Many of these disincentives to research careers—unstable research funding, financial pressures on medical institutions, and student debt—remain. Training times are lengthy. Physicians are often far removed from the research world during residency and fellowships, and re‐entry is challenging.^15^ Hall et al.^16^ estimated that it takes 15 years after earning a medical degree for physician‐scientists to receive their first NIH grant. Van Epps and Younger^17^ found that it required more than 10 years after the completion of training for emergency physician‐scientists to obtain an R01 grant from NIH. For neurologists pursuing research careers, the training requirements can span two decades after high school graduation.^18^

Other factors may also explain some of the shortfall of physician‐scientists. Ley and Hamilton identified a major gender gap in NIH grant applications.^19^ By the early 2000s, women had achieved parity with men in medical school applications, admissions, and graduation. Women and men applied in equal numbers for early career research funding, and had comparable levels of success. But despite this early success, women were far less likely than men to apply for R01 grants at the transition to independence. The NIH workforce study also found that, while women and men had equal award rates, there were substantially fewer female grant applicants at the transition to independence, and that this trend worsened over the course of a career.^4^

## 2 PROGRAMS AND INITIATIVES

Efforts to stabilize the physician‐scientist workforce have focused on a wide range of interventions. In addition to long‐standing calls to provide significantly more resources for research training, debt relief, and early career support of potential physician‐scientists,^20^, ^21^ new proposals have been offered. Ley and Hamilton recommended more effective efforts to retain women in research careers, especially strategies focused on the transition to independence.^19^ Jain et al.^21^ called for more flexible family leave policies. To compensate for decades of discrimination, inadequate mentoring, and a lack of role models, there have been proposals for augmented efforts to recruit underrepresented minorities into research careers.^4^, ^15^, ^21^, ^22^, ^23^ Others have recommended increased research opportunities during medical school, residency, and specialty training,.^16^, ^18^, ^21^, ^22^, ^24^ A number of proposals have been advanced to enhance mentoring of physician‐scientists at the individual, institutional, and national levels.^15^, ^21^, ^22^ These have included physician‐scientist career development offices at the institutional and national level^21^, ^23^ and national networks to diversify clinician investigator faculty.^16^

The NIH workforce study^4^ and others^21^, ^22^ proposed increases in the number of career development awards providing “protected time,” like NIH K Grants, so that early career physician‐scientists could launch successful research programs. Evidence suggests that mentored K Awardees were far more likely to receive R01 Awards than medical school graduates without them.^25^ Comparing a matched sample of K Awardees and unsuccessful K Applicants, Nikaj and Lund^26^ found that a mentored K Award was associated with a 24.1% increase in the likelihood of a subsequent, independent NIH award. A study of obstetrics and gynecology physician‐scientists also reported that receipt of an NIH K Award led to higher levels of subsequent NIH grant funding.^27^ Among pediatric surgeons, 63% of those with K Awards were successful in obtaining subsequent R01 Grants.^28^ Analyses of similar early career development programs, including the Doris Duke Charitable Foundation Clinical Scientist Development Award, found that program alumni were more likely than non‐alumni to have received subsequent NIH R01 Grants.^29^

Programs to foster the development of physician‐scientists have been created by federal agencies, foundations, specialty boards, and research institutions. The Intramural NIH Clinical Research Training Program provides a year‐long program of mentored research opportunity for medical and dental students. Nearly two‐thirds of those who completed the training reported that they were conducting research, and over one‐quarter of these had received subsequent NIH funding.^30^

Medical specialty groups have also established programs to encourage the career development of physician‐scientists. The American Society for Reproductive Medicine created the Clinical Research/Reproductive Scientist Training Program with support from the National Institute for Child Health and Human Development.^31^ The Society of University Surgeons developed recommendations on how to choose an initial job and identify a team of committed mentors.^32^ Several of these initiatives have demonstrated positive outcomes. An evaluation of the Norman S. Coplon Extramural Grant program for early career physician‐scientists in nephrology reported impressive results, with more than 90% of program alumni staying in academia.^33^ A training program to prepare physician‐scientists for independent careers in child psychiatry found that former trainees outperformed a control group on several outcome measures, including NIH R series grants.^34^ All but one of the 34 Pediatric Critical Care Trauma Scientist Development Fellows remained active in research, and 60% had been promoted to associate or full professor.^35^ GME research participation for surgery fellows was associated with faculty appointments and NIH grants.^36^ Incorporation of graduate degree programs into specialty or subspecialty training has been used to help clinically trained individuals develop research skills.^16^, ^18^, ^37^

There have been frequent calls to reduce training time for physician‐scientists,^15^, ^23^ and several specialty boards have created expedited “research pathways” for physicians pursuing research careers in internal medicine,^38^ pediatrics,^39^ radiology,^40^ dermatology,^41^ and pathology.^42^ Before the pathology program was initiated, Remick et al.^42^ found that pathology departments were highly successful at educating physician‐scientists, but less successful at recruiting them into research careers. Pathology had the highest percentage of MD‐PhDs, and pathologists received the highest percentage of NIH postdoctoral research training awards. But pathology departments had fewer K awards than other medical school departments. The new pathology research pathway was designed to better facilitate the transition from training to research careers.

Medical schools have also launched efforts to increase the number of physician‐scientists. In 2000, Washington University School of Medicine established the first Physician‐Scientist Training Program (PSTP) to integrate residency, fellowship, and postdoctoral training for individuals committed to careers in academic medicine.^43^ Over 30 medical schools now have PSTPs, with several reporting highly successful outcomes. The PSTP at the University of Pittsburgh Medical School found that graduates exceeded their peers in terms of publications and research funding.^44^ The University of California, Los Angeles initiated the Specialty Training and Advanced Research Program to fund protected time for trainees to pursue a graduate degree shortly before completing their specialty or subspecialty clinical training. This program combined graduate course work and research training with a subspecialty fellowship. More than 80% of the program graduates were actively conducting research. Individuals who obtained PhDs during subspecialty training were more likely to receive R01 (or equivalent funding) than those who earned a Master of Science in Clinical Research.^37^ In 2017, the Burroughs Wellcome Fund created Physician‐Scientist Institutional Awards for medical schools proposing innovative approaches to MD‐only, “late bloomer” physician‐scientist development. Five awards were made in 2018 and five more in 2019.^45^

## 3 THE CHANGING RESEARCH ENVIRONMENT

**FIGURE 1:**
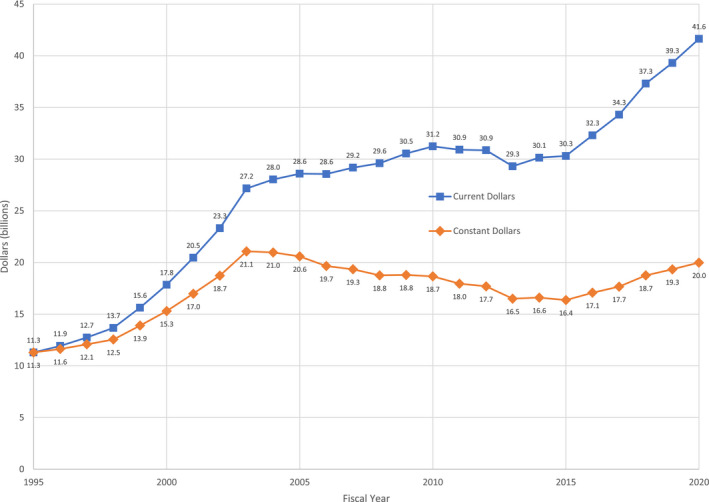
NIH budget in current and constant dollars, FY1995‐2020 (*Source*: National Institutes of Health, History of Appropriations, Fiscal Years 1982–2020)

The landscape for medical research has changed dramatically over the past decade. When the NIH and FASEB studies of physician‐scientists were conducted, the NIH was in the midst of a prolonged period of budgetary constraint. The 2013 NIH budget was $29.3 billion, only $2.1 billion above its 2003 level. In constant dollars (adjusting for inflation), NIH funding in 2013 was 21.7% below its 2003 level.^4^ By the end of the decade, however, the situation was markedly different. NIH received substantial budget increases in FY2016 though FY2020 (Figure 1). Increases of $2.0 to $3.0 billion per year raised NIH funding from $30.311 billion in 2015 to $41.437 billion in 2020, an increase of 37.4%.^46^

Broader changes in the U.S. healthcare system, including the Affordable Care Act, have altered the climate for research in academic medical centers.^4^ Increased numbers of patients on Medicaid and a decrease in Medicare reimbursement for medical services put financial pressures on hospitals, limited the availability of institutional funds to support early career researchers, and placed greater pressure on faculty to generate clinical revenue. Other factors, like the emergence of the COVID‐19 pandemic, also have had implications for the research workforce. The heightened awareness of the need for new vaccines, diagnostics, and therapies may be influencing young physicians to pursue research careers. A striking increase in medical school applications during the COVID pandemic also suggests that medical training is becoming a more popular career choice for young people in this country. In the 2021–2022 academic year, medical school applications rose by 9413 (17.8%), the largest increase ever recorded in a single year.^47^

Shifts in research funding and health care policy, along with the new programs and initiatives mentioned above, can affect the opportunities and incentives for research careers. In light of these changes, it is important to re‐examine the numbers of physician‐scientists at the various stages of training and research involvement.

## 4 DATA AND METHODS

The data used in this study were taken from administrative records maintained by NIH and surveys conducted by the Association of American Medical Colleges (AAMC) and the American Medical Association (AMA). Information on major professional activities of U.S. physicians come from the AMA Physician Masterfile and are reported in the annual volumes of *Physician Characteristics and Distribution in the U*.*S*.^48^ Print publication of this series ended in 2015, and subsequent years' data come from the AAMC *Physician Data Specialty Reports* (2016, 2018, and 2020)^49^ and tabulations purchased from the AMA contract vendor, MMS, Incorporated, in Schaumberg, Illinois. Data on physicians with full‐time faculty positions at U.S. medical schools are from the AAMC Faculty Roster.

Data on several factors known to be associated with the establishment of a research career—student interest, student debt, and faculty appointments—come from AAMC. Information on medical students' career plans comes from the *AAMC Matriculating Student Questionnaire All Schools Summary Reports* ^50^ and the *Medical School Graduation Questionnaire All Schools Summary Reports*.^51^ Statistics on medical school debt, based on the Graduation Questionnaires, are from AAMC reports.^52^

Additional information on correlates of research careers—research fellowships, loan repayment grants, career development grants, and availability of research funding—comes from NIH. Data on the NIH budget was taken from the NIH Budget Office website.^46^ Extramural and Intramural Loan Repayment Program (LRP) data come from the LRP Data and Reports Page https://dashboard.lrp.nih.gov/app/#/.^53^ Information on F32 fellowship applications, awards, and success rates are from tabulations posted on the NIH Frequently Requested Reports webpage.^54^ Other NIH data are from the *NIH Data Book* ^55^ and the individual publications cited below.

## 5 RESULTS

### 5.1 Matriculating and graduating medical students' interest in research careers

The decision to pursue a career as a physician‐scientist can be made at many points in an individual's training. For some, it is the primary reason for pursuing a medical degree, and opportunities for research training are available at the earliest stages of medical education. Dual degree programs, enabling individuals to earn both a medical degree and a research degree, were created specifically for those medical school applicants who aspired to research careers. Highly competitive MD‐PhD programs, like the NIH‐funded Medical Scientist Training Program (MSTP), provide an opportunity for individuals with early identified interest in research to develop the research and clinical knowledge necessary to further their goals. From 1993–2013, MD‐PhD program enrollments more than doubled, while the total number of medical school matriculants grew by 17%.^56^ MD‐PhD enrollments have continued to grow, with the total number of medical students in MD‐PhD programs rising from 5010 in the 2011–2012 school year to 5830 in 2020–2021.^57^

MD‐PhD programs train some—but not all—physician‐scientists. Many physician‐scientists earn their MD and PhD degrees consecutively, not simultaneously.^4^ For many, the desire to conduct research emerges during medical school and residency, perhaps as a result of exposure to unmet clinical needs and limitations of current therapies. These are the so‐called “late bloomers.” Until recently, MDs without PhD degrees comprised the largest number of physicians with T32 training grant positions, F32 research fellowships, K awards, and R01 research grants.^10^ “MD‐only” investigators still comprise about half of the NIH‐funded physician‐scientist workforce, and their success in the NIH granting pool is comparable to PhDs and MD‐PhDs.^4^, ^19^

In 2020, 63.4% of matriculating medical students reported plans to participate in research during their career.^50^ This was a slight increase from 2015, when this fraction was 61.1 (Table 1). Of those reporting plans to participate in research, the fraction planning to be “full‐time” or “significantly” involved in research rose from 42.5% in 2015 to 47.1% in 2020, with the majority of the increase coming in 2017–2020, years of large NIH budget increases. This is consistent with an earlier study in which the percentage of matriculating medical students with significant or exclusive interest in a research career rose sharply during the 1998–2003 “doubling” of the NIH budget.^10^

**TABLE 1:** Student interest in research careers, research activities in medical school, and indebtedness of medical school graduates (2011–2020)

	2011	2012	2013	2014	2015	2016	2017	2018	2019	2020
Research Career Plans^a^										
Matriculating students with plans for research					61.1%	61.5%	62.2%	61.2%	61.9%	63.4%
If yes, full‐time or significant participation					42.5%	42.8%	42.9%	46.2%	45.8%	47.1%
Graduating students with plans for research					59.7%	52.2%	52.9%	51.3%	51.8%	51.0%
If yes, full‐time or significant participation					44.7%	47.3%	46.6%	47.5%	47.7%	47.8%
Research activities during medical school^b^										
Research project with faculty member	66.3%	68.1%	68.2%	69.3%	69.4%	74.1%	77.3%	78.8%	80.9%	82.5%
Authorship (sole or joint) of a research paper submitted for publication	40.6%	41.8%	41.7%	42.0%	47.8%	46.5%	48.6%	50.5%	54.0%	55.1%
Authorship (sole or joint) of a peer reviewed oral or poster presentation				43.6%	52.5%	50.3%	53.3%	56.7%	60.6%	63.4%
Medical School Debt of U.S. Medical School Graduates^c^										
Percentage with debt	86%	86%	86%	84%	81%	76%	75%	75%	73%	73%
Median Education Debt in 2019 Dollars	$184 000	$189 000	$192 000	$194 000	$197 000	$202 000	$200 000	$203 000	$200 000	

^a^ AAMC Matriculating Student Questionnaire All Schools Summary Reports and Medical School Graduation Questionnaire All Schools Summary Reports, 2012–2020.

^b^ AAMC Medical School Graduation Questionnaire All Schools Summary Reports 2015, 2019, and 2021.

^c^ James “Jay” Youngclaus and Julie A Fresne, Physician Education Debt and the Cost to Attend Medical School 2020 Update, AAMC, Washington, DC.

Unlike the matriculating students, the research plans of graduating medical students did not rise when the NIH budget grew from 2016–2020. In 2016, 52.2% of the graduates had interest in research, and it remained at this level through 2020 (Table 1). In addition, the percentage of graduating students reporting plans to participate in research during their careers was lower than that of their matriculating counterparts. Over the course of their medical education, student aspirations to actively engage in research declined. Among 2020 graduates, for example, 51.0% planned to participate in research during their careers, whereas 61.5% of the 2016 matriculating students reported similar plans. This is a reversal of the pattern found in the 1990s and 2000s, when graduating students reported higher levels of interest in research careers than matriculating students.^10^ Although the reason for this change is not clear, we speculate that the diminishing interest in research during medical school may be a result of recent curriculum reforms that place increased emphasis on clinical decision‐making, and a decreased emphasis on foundations in basic science, which may be having a negative effect on the production of physician‐scientists.^58^

### 5.2 Research experience during medical school

The percentage of graduating medical students reporting research activity during medical school has been rising in recent years. In 2020, 82.5% of the graduating medical students reported participating in a research project with a faculty member (Table 1). This reflects a steady increase since 2011, when 66.3% had a research experience. During this same period, the number of graduating medical students with sole or joint authorship of a research paper rose from 40.6% to 55.1%. Authorship of a peer‐reviewed oral or poster presentation showed similar growth. Jeffe and Andriole^25^ found that graduates with research electives during medical school and authorship of a peer‐reviewed paper or poster were more likely than their peers to successfully compete for postdoctoral research fellowships (F32), mentored career development (K) awards, and R01 research grants from NIH.

### 5.3 Indebtedness of U.S. medical school graduates

Rosenberg postulated that financial pressures arising from medical school debt discouraged graduates from undertaking an extended program of research training or incurring the economic risks of a research career and instead led them to pursue higher paying careers in private practice.^7^ Zemlo et al. found that a rising level of student debt in the 1990s was correlated with a declining proportion of physicians choosing research careers.^8^

From 1982 through 2011, the mean indebtedness of medical school graduates tripled, after adjustment for inflation.^10^ In the second decade of the 21st Century, medical education remains an expensive undertaking, but the percentage of medical school graduates with educational debt has declined slightly. In 2020, the percentage of medical school graduates with education debt was 73%, a decrease from 86% in 2011 (Table 1). During this period, the median debt level grew, but at a slower rate than in previous decades. The median debt of medical school graduates with debt, when adjusted for inflation, increased from $184 000 in 2011 to $200 000 in 2019 (2019 dollars).^52^

### 5.4 NIH loan repayment programs

In response to the widely accepted idea that medical school debt may discourage research careers, NIH introduced its extramural Loan Repayment Program (LRP) in 2004 to assist recent graduates' pursuit of research careers. The LRP now repays up to $50 000 annually (for up to three years) of a researcher's qualified educational debt in return for a commitment to engage in NIH mission‐relevant research. The program is, however, restricted to researchers in specific fields: clinical research, pediatric research, health disparities research, contraception and infertility research, clinical research for individuals from disadvantaged backgrounds, and research in emerging areas critical to human health. Over the past two decades, the NIH loan repayment program has paid off the educational debts of several thousand physicians, and has undoubtedly made an important contribution to the number of physician‐scientists in the workforce.

LRP began in 2004 with 1407 awards to extramural scientists. An intramural program was created for scientists on NIH's Bethesda campus in 1989 and funded 89 awards in its inaugural year. The number of Extramural LRP awards increased during the early years of the program, reaching 1604 in 2009. But when NIH budget growth ended in the second decade of the current century, the number of Extramural LRP awards began to decline, and Extramural LRP awards fell from 1572 in 2011 to 1262 in 2020. There was a similar decline of about 20% in Intramural LRP awards.^53^ When the NIH budget rebounded in 2016–2020, no concurrent rise in either Extramural or Intramural LRP awards occurred.

Approximately half of the Extramural LRP awards have been awarded to physicians. In 2020, MDs comprised 43.3% of the awardees, with MD‐PhDs making up 6.7%. Physicians comprised an even larger fraction of the Intramural LRP awardees, with the vast majority of the intramural awards (74.1%) going to MDs and another 5.9% to MD‐PhDs.^53^ Publicly available data for LRP applicants and awards do not include sex or under‐represented minority status by degree, so we do not know whether specific subgroups of LRP applicants are uniquely affected by these trends.

**FIGURE 2:**
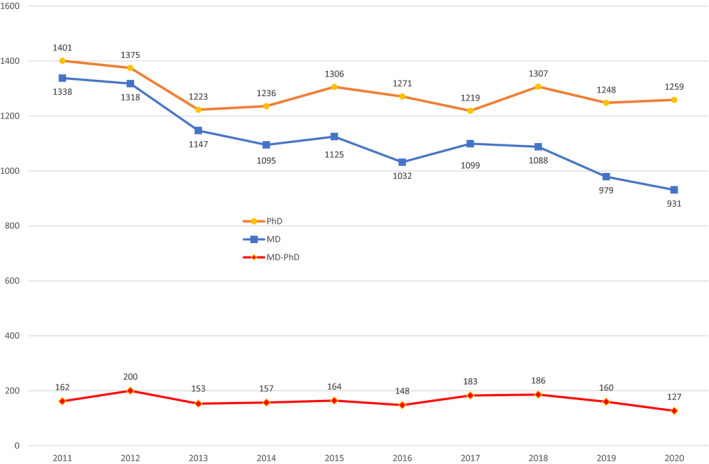
NIH Extramural Loan Repayment Program Applications by Degree, 2011–2020 (*Source*: National Institutes of Health. Loan Repayment Program Dashboard https://dashboard.lrp.nih.gov/app/#/, 2021 accessed July 1, 2021)

The declining number of Extramural LRP awards is associated with decreased applications (Figure 2). The decline in applications for Extramural LRP awards was steady throughout the decade, and did not rise when the NIH research funding grew significantly in 2016 through 2020. The number of applications from MDs for Extramural LRP awards steadily declined from 1338 in 2011 to 931 in 2020, a decrease of 30.4%. MD‐PhD applications declined by 21.6%, falling from 162 to 127. For PhDs, applications declined only 10% during the same period (from 1401 to 1259). Applications for Intramural LRP awards remained steady over the past decade, averaging approximately 36 new and 46 renewal applications per year.

### 5.5 Postdoctoral fellowships

**FIGURE 3:**
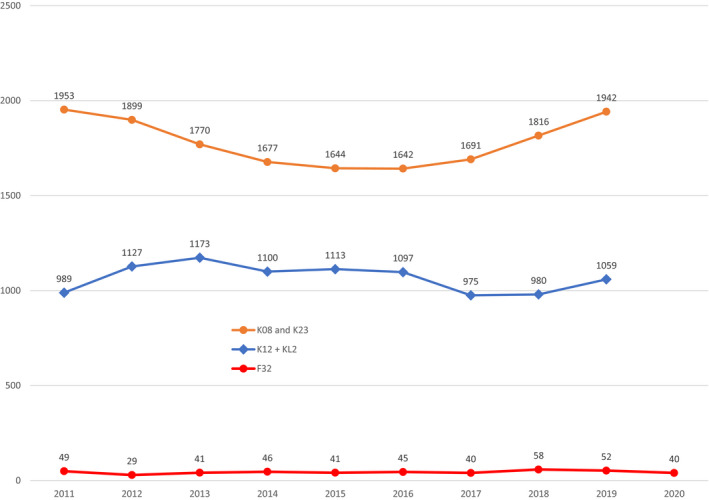
NIH F32 Fellowship Awards and K08, K23, K12, and KL2 Career Development Awards for MDs and MD‐PhDs, 2011–2020 (*Source*: https://report.nih.gov/nihdatabook/report/223 and https://report.nih.gov/nihdatabook/report/299)

Postdoctoral research training is a necessary part of a scientist's preparation for a research career, and for decades, observers have used data on postdoctoral research training to measure the status of the training pipeline.^6^, ^7^, ^8^ One of the most prestigious and competitive vehicles for postdoctoral research training is the NIH Ruth L. Kirschstein Postdoctoral Individual National Research Service Award, or F32. During the late 1980s through the early 1990s, approximately 300 MDs received F32 grants each year.^8^ F32 awards to MDs dropped precipitously in late 1990s and early 2000s. In 2002, when the NIH budget was in its fourth year of double‐digit increases, only 73 MDs and 20 MD‐PhDs received F32 awards.^10^ By 2011, 35 MDs and 14 MD‐PhDs received these awards. Since that time, the number of F32 awards to MDs and MD‐PhDs has remained at approximately 50 per year (Figure 3).

The decline in postdoctoral fellowship awards to physicians reflects the declining number of applications. In the aftermath of the 1998–2003 doubling of the NIH budget, over 200 MDs and over 50 MD‐PhDs applied for the F32 fellowships. In 2011, there were 163 physician applicants, and by 2020, there were only 134. Success rates for physicians, however, remained around 30%, comparable to other applicants.

In addition to F32 individual postdoctoral fellowships, there are institutional awards that fund postdoctoral research training. The NIH Ruth L. Kirschstein Institutional National Research Service Award (T32) enables institutions to recruit individuals for research training. From 1985 through 1992, approximately 1600 MDs per year held positions on institutional postdoctoral training grants.^10^ By 1997 the number fell to fewer than 1200. When NIH funding began to grow in 1998, so did the number of MDs on T32 grants. The number of T32 positions held by MDs reached 1700 in 2003 and remained at that level until 2011, when T32 positions declined to 1111. The current number of MDs on T32 grants, however, is no longer reported by the NIH.

### 5.6 Research career development awards (K grants)

Career development awards provide protected time for early career scientists to develop a research program, and the goal of these awards is to bring candidates to the point where they are able to conduct research independently with their own research support. NIH offers a series of individual and institutional Research Career Development Awards. The number of individual K Awards rose from 2312 in 1998 to 4334 in 2007. When the NIH research budget failed to grow in subsequent years, the number of individual K Awards declined, and in 2016 there were only 3671 awardees. But with rising levels of research funding after 2015, the number of individual K Awards rose again, reaching 4492 in 2020.^55^

There are a variety of individual K programs targeting specific career stages and research areas. The largest programs are the K01 Mentored Research Scientist Career Development Award, K08 Mentored Clinical Scientist Research Career Development Award, K23 Mentored Patient‐Oriented Research Career Development Award, and K99 Pathway to Independence Award. While MDs can apply for any of these awards, they are most often supported by the K08 and K23 mechanisms. These two categories comprise approximately half of the K Awards.

NIH no longer posts data on individual K Awards by recipients' degree, so it is unclear how many MDs are now receiving K Awards. But a lower bound estimate of the number of physicians receiving K Awards can be derived from statistics on those programs targeted to physicians. The number of individuals supported on K08 and K23 Awards reached a peak of 2233 in 2005, shortly after the 1998–2003 “doubling” of the NIH budget. After several years in which the NIH budget failed to keep pace with rising research costs, the number of individuals on K08 and K23 Grants declined, reaching a low of 1642 in 2016 (Figure 3). With rising funding for NIH in the next four years, the total number of K08 and K23 Awards increased and reached 2070 in 2020 (520 new awards and 1550 noncompeting continuations).^55^

In addition to individual K Awards, NIH supports institutional programs that provide career development training for physician‐scientists. The Clinical Scientist Institutional Career Development Program Award (K12) is an institutional grant to prepare newly‐trained physicians who have made a commitment to independent research careers. The Mentored Career Development Award (KL2) supports newly trained clinicians appointed by an institution for activities promoting development of clinical or translational research careers. Approximately one thousand individuals were supported on institutional K Awards each year from 2011 to 2020 (Figure 3). Taken as a whole, at least 3000 physicians were supported on career development awards each year from 2011 through 2020.

### 5.7 Number of physician‐scientists

Physician‐scientists work in a variety of settings including medical schools, research universities, teaching hospitals, pharmaceutical companies, biotechnology firms, contract research organizations, free‐standing research institutes, government agencies, voluntary health agencies, and private practice. In each of these settings, the amount of time individuals devote to research is variable, and thus even an exhaustive tally of individuals in specific employment settings cannot capture the total amount of research conducted by physicians.

Previous studies have used different measures to assess the number of physician‐scientists. The most commonly used indices are medical school faculty positions, surveys of major professional activity, and NIH research grants. While none of these measures captures the full range of research involvement by physicians, each can provide a useful perspective on change over time, since these measurements have been made in the same way for decades.

#### 5.7.1 Medical school faculty

The number of physicians on medical school faculties is an indicator of physicians in a major research environment. Medical school faculty members receive approximately half of the NIH research funding,^59^ and analysts have used full‐time medical school faculty positions as a research career outcome measure.^56^ There is, however, a wide range of research involvement among physicians in academic settings. A recent survey of MD‐PhDs, for example, found that some faculty members devote a substantial portion of their effort to research, while others are primarily involved in patient care.^60^

Between 2011 and 2020, the number of physicians (MD and MD‐PhD) holding full‐time faculty positions at U.S. medical schools rose by 28.4%. There are a variety of reasons for this growth, including the formation of new medical schools, growing faculty numbers at established medical schools, and, in all likelihood, addition of new hospital affiliations and expansion of medical faculty practice plans. Almost all of the growth was in clinical departments, and involved individuals with MD degrees only. From 2011–2020, there was almost no growth in basic science departments, and only slight growth in the number of faculty members with both MD and PhD degrees (Table 2). In 2011, there were 95 188 MD faculty in clinical departments. By 2020, there were 124 946, an increase of 31.3%. The number of MD‐PhDs in clinical departments grew at a slower rate (12.3%), rising from 11 044 to 12 399 during the same period. MD faculty in basic science departments rose from 1824 in 2011 to 2051 in 2020, an increase of 227 positions (12.4%), while the number of MD‐PhD faculty in basic science departments declined from 1735 to 1587. There has been an increase in the number of physicians in academic medicine, but the vast majority of this growth was in clinical departments—with limited growth in basic science departments.

**TABLE 2:** Full‐time U.S. medical school faculty with MD and MD‐PhD degrees by department type (2011–2020)

Year	Basic science departments		Clinical science departments	
	MD degree	MD‐PhD or MD and other doctoral degree	MD degree	MD‐PhD or MD and other doctoral degree
2011	1824	1735	95 188	11 044
2012	1864	1775	98 072	11 238
2013	1865	1775	102 133	11 452
2014	1896	1763	106 538	11 677
2015	2027	1790	110 448	11 944
2016	1950	1721	113 839	12 206
2017	1981	1727	116 371	12 371
2018	1983	1715	118 198	12 496
2019	2028	1656	121 580	12 384
2020	2051	1587	124 946	12 399
Percent Change 2011–2020	12.4%	−8.5%	31.3%	12.3%

#### 5.7.2 Major professional activity

Another measure of physician research participation is the annual AMA survey of professional activity. The survey frame covers individuals enrolling in U.S. medical schools and foreign medical graduates when they enter the U.S. The Physicians Practice Arrangements Questionnaire asks physicians about their major professional activity, and the medical research response category includes both funded and unfunded medical research. The major advantage of this survey is its continuity and ability to assess change over time. Administered annually since the early 1980s, this survey provides a longitudinal measure of change in the number of physicians with a primary involvement in research. It contains information on AMA members and non‐members. But as an index of physician research activity, it has its limitations. Survey nonresponse may result in an undercount of the number of researchers. Moreover, by focusing on “major” professional activity, this measure may not capture part‐time research involvement and collaboration on research teams.

In 2011, 13 557 U.S. physicians reported research as their major professional activity; by 2020, there were 12 289, a decrease of 1268 physician‐scientists (9.4%: Table 3). Over the same period, the total number of actively employed physicians rose from 869 623 to 937 035. As a result, the percentage of U.S. physicians reporting research as their major professional activity declined from 1.6% in 2011 to 1.3% in 2020. Despite numerous efforts to increase the population of physician‐scientists, there has been a decrease, both absolute and relative, in the number of U.S. physicians reporting research as their major professional activity in the past decade.

**TABLE 3:** U.S. physicians reporting research as major professional activity (2011–2020)

Year	Active physicians^a^	Research	Percentage with research as major activity
2011	869 623	13 557	1.6%
2012	883 650	13 481	1.5%
2013	829 962	13 452	1.6%
2014	849 271	13 228	1.6%
2015	860 939	13 123	1.5%
2016	876 600	12 837	1.5%
2017	892 856	12 838	1.4%
2018	913 987	12 884	1.4%
2019	938 980	12 632	1.3%
2020	937 035	12 289	1.3%

^a^ Total number of physicians minus number of inactive physicians and those with unknown addresses.

#### 5.7.3 NIH research grants

For decades, beginning with Wyngaarden's seminal study,^6^ the number of NIH research grants to individuals with MD degrees has been used to assess the physician‐scientist workforce. While NIH grants are not the only source of support for physician‐scientists,^61^ NIH is the largest single funder of biomedical research in the U.S. For decades, receipt of a first R01‐Equivalent grant has been used to measure the number of new physician‐scientists entering the research workforce.^2^, ^4^, ^10^ R01 (or equivalent) grants, the major mechanism used by the NIH to fund multi‐year investigations, confer recognition that the recipient has identified a significant research problem and proposed a viable mechanism to address it. Unfortunately, the NIH is no longer posting data on research grant applications and awards by degree and sex. At one point, in response to a recommendation of the Physician‐Scientist Workforce Working Group, NIH created an online dashboard with this information,^62^ but it is no longer active.

A limited perspective on awards to physician‐scientists, however, can be found in a recent blog post on the *NIH Extramural Nexus*.^63^ The number of individuals receiving their first NIH R01‐Equivalent awards declined from 1877 in 2010 to 1523 in 2015, but rose to 2243 in 2020 (Table 4). Year‐over‐year data for these awards are not currently available, making trends difficult to assess accurately. The increased number of first R01 awards may have been a result of new NIH policies designed to help early career investigators. MDs appear to have been particularly successful under the new conditions. First R01‐Equivalent awards to MDs rose from 219 in 2015 to 344 in 2020, an increase of 57.1%, a higher rate of growth than that of MD‐PhDs and PhDs. Based on these two data points, these findings suggest improving circumstances for physician‐scientists. Additional data are required, however, to confidently conclude that a major change has taken place.

**TABLE 4:** First NIH R01 award for principal investigators by degree for FY2010, 2015, and 2020

Year	MD	MD‐PhD	PhD	Total
2010	282	216	1379	1877
2015	219	148	1156	1523
2020	344	215	1684	2243

### 5.8 Age at first R01‐Equivalent grant

Increasingly long training times and growing requirements for specialty and subspecialty certification delay the onset of physician‐scientists' research careers, and consequently physicians are older than other scientists when they begin their independent research careers. For MDs, the average age at first R01‐Equivalent Grant was 45.1 years in 2011 (Table 5). The average age for MD‐PhDs was 44.3 years, and the average age for PhDs was 42.4 years. Since they are older when they start their research careers, physician‐scientists will necessarily have shorter active careers.

**TABLE 5:** Average Age and Degree of First‐Time NIH R01‐Equivalent Investigators (2011–2020)

Fiscal Year	MD	MD‐PhD	PhD
2011	45.1	44.3	42.4
2012	44.7	44.7	42.2
2013	45.2	43.6	42.1
2014	45.0	44.8	42.0
2015	44.9	44.9	42.2
2016	45.3	45.2	42.6
2017	44.8	45.4	42.4
2018	45.8	45.5	42.3
2019	46.1	45.5	42.2
2020	46.1	45.5	42.5

Note

The definition of First‐Time investigator has changed over time, and data reflect investigator policies that were in place during those years. Data produced by the division of statistical analysis and reporting—OERStats@mail.nih.gov

By 2020, the mean age of MDs was 46.1 years when they received their first R01 grant, an increase of one full year since 2011. MD‐PhDs experienced a similar increase, with their average age at first R01 rising from 44.3 years in 2011 to 45.5 years in 2020. During the same time period, the average age at first R01‐Equivalent grant for PhDs remained stable. Therefore, there is no evidence that new programs created by specialty boards to expedite the certification of physician‐scientists has reduced the average time needed to initiate a successful research career. It may be the case, however, that the average age at first R01 would have been even higher for physicians in the absence of the expedited specialty certification tracks.

## 6 DISCUSSION

Over the past decade, there have been numerous efforts to increase the number of physician‐scientists. Following the release of the NIH Physician‐scientist Workforce Report,^4^ organizations and institutions created new programs to foster training and support early career development of physician‐scientists. These programs created more efficient career paths, offered intensive mentoring, and expanded opportunities for protected research time.

Several other recent factors would be expected to increase the number of physician‐scientists. NIH funding increased from 2016–2020, and this has been historically associated with rising levels of research career intentions among matriculating medical school students, and increased physician participation in research training programs. The number of students entering MD‐PhD programs has been slowly rising, and most of these individuals become academic physician‐scientists. The percentage of medical school graduates reporting elective research activities or papers submitted for publication has risen steadily over the past decade, and these activities are correlated with successful applications for fellowships, career development awards, and research grants. NIH career development awards (K grants) increased as the NIH research budget began to grow after 2015, and K grantees are more successful as applicants for subsequent NIH R01‐Equivalent funding.^26^, ^29^ There has also been a steady increase in medical school faculty members with MD degrees, a finding that suggests increased physician participation in research.

There are, however, several countervailing trends that suggest growing challenges for the physician‐scientist pathway. These negative trends should be of concern to policymakers, and those charged with oversight of the future of biomedical research in the U.S. Specifically, interest in research careers, which has long increased while students go through medical school, is now lower at graduation than at matriculation. The number of F32 postdoctoral research fellowship applications from MDs and the number of F32 awards to MDs are now far below historic levels. The number of career development awards targeted to physicians has not increased in the past decade. Efforts have been made by certification boards to create special research tracks, and NIH has instituted policies to aid early career investigators. Despite these innovations, the average age at receipt of a first R01‐Equivalent grant continues to rise for MDs and MD‐PhDs. It took more than a year longer for physicians to obtain an R01 in 2020 than it did in 2011. For PhDs, the average age at first R01‐Equivalent grant has remained constant since 2011.

Although there has been a slight reduction in the fraction of medical school graduates with student debt over the past decade, the median debt burden for most graduating students is still nearly $200  000. However, LRP applications from MDs have declined by more than 30% from 2011–2020. Since most students still have enormous debt burdens at graduation, we find the decreasing physician participation in the NIH LRP to be one of the most worrisome findings of this study. The LRP is often the first NIH grant application for young physician‐scientists, and it may therefore be the earliest indicator of the stability of the pathway.^19^ The LRP is crucial for the health of the “late‐bloomer” pathway, since MDs continue to bear the full costs of their medical education. The MD population has traditionally represented the majority of physician‐scientists, and clearly needs to be protected, since the number of new MD‐PhD graduates each year cannot sustain the estimated 500–1000 new physician‐scientists needed each year for stability of the workforce.^64^ MDs also tend to have different specialties and subspecialties than MD‐PhDs,^60^, ^65^ and the loss of MD physician‐scientists will restrict the population of physician‐scientists to a narrower range of fields and research interests. The factors contributing to the decline in LRP applications are essential to understand, but the limited information available from the LRP Dashboard makes the cause unclear at this writing.

Of considerable concern, the AMA's metric of MDs reporting research as their major professional activity declined by over nine percent from 2011–2020—representing a decline of more than 1200 individuals in this workforce. However, the number of MDs in academic medicine has grown, and the number of physicians receiving their first R01 award in 2020 was 57% larger than the comparable figure for 2015 (with limited data to confirm that this represents a real trend). Weighing all of these indicators, it is difficult for us to draw definitive conclusions about the current status of the physician‐scientist workforce.

What is clear, however, is that there is even less information on this important subject today than there was a decade ago. Data on the number of physicians supported on T32 institutional training grants, receiving K01 Mentored Research Career Development Awards, and awarded R01‐Equivalent research grants are currently unavailable. Posting more information by researchers' degrees, under‐represented minority status, and sex are critical to understand what is happening to the workforce. More information on the composition of applicant and awardee pools is needed. NIH should restore the dashboard tool with data on all funded scientists, since this information is essential for prospective trainees, training program directors, and policymakers. While information on early career applicants and awardees is crucial, it is also essential to track individual investigators over time to discern when they enter and leave the NIH‐funded workforce. The full range of data collected by the Physician‐Scientist Workforce Working Group is needed to make informed policy decisions, and to assess their impact. We therefore call on the NIH leadership to reconvene the Physician‐Scientist Working Group (which met last in 2013) to evaluate the successes and failures of the first report, and to consider remedial and/or new recommendations pertinent to this vital workforce, which performs a unique and essential service to the nation.

## FUNDING INFORMATION

This work was supported by the Doris Duke Charitable Foundation. The views expressed in this paper, however, are those of the authors and may not represent those of the Doris Duke Charitable Foundation. TJL was supported by the Barnes Jewish Hospital Foundation and the Lewis T. and Rosalind B. Apple Chair in Oncology.

## DISCLOSURES

The authors have no conflicts of interest.

## AUTHOR CONTRIBUTIONS

Howard H. Garrison designed the research and prepared the initial draft of the paper; Timothy J. Ley contributed to the analysis and revision of the manuscript.

## Data Availability

The statistical data used in this study were originally collected by the National Institutes of Health (NIH), the Association of American Medical Colleges (AAMC), and the American Medical Association (AMA). All tabulations and graphics were generated by the authors using Microsoft Excel. The specific sources for the data used in this research are cited in the methods section and in the tables and figures used in the report.
